# Challenges and considerations in prehospital triage for traumatic brain injury patients

**DOI:** 10.1186/s13049-024-01244-8

**Published:** 2024-09-06

**Authors:** Şebnem Zeynep Eke Kurt

**Affiliations:** Istanbul, Turkey

**To the Editor**,

I read with great interest the article entitled "Patients suffering traumatic brain injury: patient characteristics, prehospital triage, primary referral and mortality—A population-based follow-up study" by Seidenfaden et al. [1]. This comprehensive population-based study provides valuable insights into triage pathways, patient characteristics, and mortality outcomes for patients with traumatic brain injury (TBI) in Denmark.

The methodology used by Seidenfaden et al., which includes a large retrospective cohort of 5,257 TBI patients over a 2 year period, is commendable for its scope and use of linked national registries. The study highlights important differences in triage patterns and outcomes based on the mode of entry into the healthcare system ( General Practitioner/Health Care Provider vs. 112 call).

However, several issues warrant further discussion and clarification:

First, while the registry-based design provides a wealth of data, it inherently limits the clinical information available. The authors acknowledge this limitation, but it would be beneficial to elaborate on how the lack of key clinical variables such as Glasgow Coma Scale scores, blood pressure, and injury severity scores affects the interpretation of their results, particularly regarding mortality comparisons [2].

The finding that patients presenting via 112 calls were more likely to be triaged to the highest level of prehospital response and referred directly to specialized centers is intriguing. However, the study could be strengthened by a more detailed analysis of the factors influencing these triage decisions and their appropriateness [3].

The authors found no significant difference in adjusted 30 day or 1 year mortality between patients referred to regional hospitals and those referred to specialized centers. This contradicts some previous studies and guidelines that recommend direct transport to neurosurgical facilities. It would be valuable for the authors to discuss possible reasons for this discrepancy, such as improvements in prehospital care or early local hospital management [4].

The identification of subdural hemorrhage as the most common intracranial lesion is consistent with other studies. However, the study could be improved by a more detailed analysis of outcomes based on specific lesion types and severity [5].

The study provides important insights into the epidemiology and management of TBI patients in Denmark. It highlights the need for further research to optimize triage tools and referral patterns. The results suggest that the current system of initial assessment in regional hospitals may be appropriate in many cases, challenging assumptions about the universal benefit of direct referral to specialized centers.

In conclusion, Seidenfaden et al. have made a significant contribution to our understanding of TBI patient characteristics, triage patterns, and outcomes in a Scandinavian healthcare system. Their findings provide a basis for future research and potential improvements in clinical practice. We hope that these discussions will encourage more comprehensive studies, including detailed clinical data, to further refine TBI management strategies.

## Data Availability

No datasets were generated or analysed during the current study.
